# Krüppel-Like Factor 4 and Its Activator APTO-253 Induce NOXA-Mediated, p53-Independent Apoptosis in Triple-Negative Breast Cancer Cells

**DOI:** 10.3390/genes12040539

**Published:** 2021-04-08

**Authors:** Wataru Nakajima, Kai Miyazaki, Yumi Asano, Satoshi Kubota, Nobuyuki Tanaka

**Affiliations:** Department of Molecular Oncology, Institute for Advanced Medical Sciences, Nippon Medical School, Tokyo 113-0033, Japan; nakaji@nms.ac.jp (W.N.); s16-098mk@nms.ac.jp (K.M.); yumi-nit@nms.ac.jp (Y.A.); gatpmw239@gmail.com (S.K.)

**Keywords:** NOXA, KLF4, transcriptional activation, APTO-253, p53, apoptosis, TNBC

## Abstract

Inducing apoptosis is an effective treatment for cancer. Conventional cytotoxic anticancer agents induce apoptosis primarily through activation of tumor suppressor p53 by causing DNA damage and the resulting regulation of B-cell leukemia/lymphoma-2 (BCL-2) family proteins. Therefore, the effects of these agents are limited in cancers where p53 loss-of-function mutations are common, such as triple-negative breast cancer (TNBC). Here, we demonstrate that ultraviolet (UV) light-induced p53-independent transcriptional activation of NOXA, a proapoptotic factor in the BCL-2 family, results in apoptosis induction. This UV light-induced NOXA expression was triggered by extracellular signal-regulated kinase (ERK) activity. Moreover, we identified the specific UV light-inducible DNA element of the NOXA promoter and found that this sequence is responsible for transcription factor Krüppel-like factor 4 (KLF4)-mediated induction. In p53-mutated TNBC cells, inhibition of KLF4 by RNA interference reduced NOXA expression. Furthermore, treatment of TNBC cells with a KLF4-inducing small compound, APTO-253, resulted in the induction of NOXA expression and NOXA-mediated apoptosis. Therefore, our results help to clarify the molecular mechanism of DNA damage-induced apoptosis and provide support for a possible treatment method for p53-mutated cancers.

## 1. Introduction

Evasion of apoptosis is one of the hallmarks of cancer and a major mechanism for oncogenesis, tumor growth, and cellular acquisition of resistance to chemotherapy [1,2]. Indeed, cancer cells can develop a variety of strategies to circumvent apoptosis, such as through enhanced expression of antiapoptotic factors or decreased expression and/or loss-of-function of proapoptotic factors [3]. In this context, resistance to apoptosis by loss of tumor suppressor p53 function is critical for tumor development [4]. In response to various cellular stressors, such as DNA damage, p53 activates the expression of genes that regulate cell cycle progression, apoptosis, DNA repair, and cellular metabolism [4]. Moreover, replication stress evoked by oncogene activation will trigger apoptosis or cell cycle arrest, resulting in the elimination of such cells [5]. Therefore, by the loss or aberrant functioning of p53, insufficient elimination of cells containing DNA damage or oncogene activation can lead to cancer development. Evasion of apoptosis by p53 mutations plays an important role in resistance to chemotherapeutic drugs, especially cytotoxic agents that induce DNA damage, such as cisplatin, doxorubicin, and paclitaxel [6]. Thus, elucidating the regulatory mechanisms that control apoptosis in cancer cells is important for the development of strategies for cancer prevention and treatment.

The B-cell leukemia/lymphoma-2 (BCL-2) family of proteins consists of critical regulators of apoptosis regulation in mitochondria [3]. Among them, the proapoptotic multidomain members BCL-2-associated X protein (BAX) and BCL2-antagonist/killer 1 (BAK) function as apoptosis executers in mitochondria, and gene knockout studies have revealed that BAX and BAK are essential inducers of p53-mediated apoptosis [7]. In contrast, antiapoptotic multidomain proteins BCL-2, B-cell lymphoma-extra large (BCL-XL), and myeloid cell leukemia-1 (MCL-1) inhibit BAX/BAK-mediated apoptosis. In response to various apoptosis-inducing signals, another BCL-2 subfamily, BCL-2 homology 3 (BH3)-only proteins, such as BCL-2 interacting mediator of cell death (BIM), BH3 interacting domain death agonist (BID), and MCL-1 ubiquitin ligase E3 (MULE), can induce apoptosis by inhibiting antiapoptotic BCL-2 family members or activating BAX and BAK [3]. We previously identified the p53-inducible gene *NOXA* [8] and found—using gene knockout mice experiments—that NOXA belongs to the BH3-only subfamily and regulates p53-dependent apoptosis [9]. Subsequently, another p53-inducible BH3-only protein, p53 upregulated modulator of apoptosis (PUMA), was also identified [10,11]. Moreover, in the context of p53-mediated tumor suppression, we found that NOXA and PUMA synergistically induce apoptosis in cancer cells [12,13]. Accumulating evidence has suggested that NOXA is an important regulator in mediating the cytotoxic effects of anticancer agents, and that cancer cells exert several strategies to counteract NOXA for their survival [14]. For example, proteasome-mediated degradation of NOXA is enhanced in chemotherapeutic-resistant cancer cells [15] and histone deacetylase (HDAC) inhibitors can reactivate epigenetically silenced *NOXA* gene expression, which results in the induction of apoptosis [16,17]. It has been shown that HDAC inhibitors promote NOXA-mediated MCL-1 degradation and induce apoptosis of triple-negative breast cancer (TNBC) cells [18]. TNBC cells lack expression of estrogen receptor (ER), progesterone receptor (PR), and human epidermal growth factor receptor type 2 (HER2) [19]. TNBC tumors have a high frequency of p53 mutations (about 80%) [20]. Due to the lack of therapeutic targets such as ER, PR, and HER2, the high p53 mutation rate, and a high tendency for metastasis, TNBC patients tend to have a poorer prognosis [19]. Therefore, the development of effective TNBC treatment strategies has become an important clinical need.

Various methods have been developed to effectively induce apoptosis in cancer cells with p53 dysfunction. For example, these include mutant p53 re-activating agents and inhibitors of p53 E3 ubiquitin ligase MDM2 [21,22]. Furthermore, p53-independent induction of BH3-only proteins by anticancer agents is advantageous for treating p53-mutated cancers. Indeed, p53-independent induction of BIM has been shown to induce apoptosis via several anticancer drugs, for example, tyrosine kinase inhibitors, imanitib and gefitinib, and a proteasome inhibitor, bortezomib [23]. Therefore, by activating a transcription factor that positively regulates the gene encoding, a BH3-only protein can be an effective treatment for cancer cells with a p53 gene mutation. However, currently, there are no cancer therapeutic agents that activate transcription factors of proapoptotic proteins to induce apoptosis, except for p53 activation drugs [21,22].

Here, we found that ultraviolet (UV) light induced p53-inedependent expression of NOXA, and that this induction was regulated by the transcription factor Krüppel-like factor 4 (KLF4). Moreover, we found that the small compound APTO-253 [24,25], which induces KLF4 function, promotes NOXA expression and NOXA-dependent apoptosis in TNBC cells. These results reveal a novel mechanism of apoptosis induction by KLF-4 and provide the possibility of a new therapeutic method for TNBC.

## 2. Materials and Methods

### 2.1. Cell Lines and Cell Culture

Human TNBC cell lines MBA-MB-468, MDA-MB-231, HCC38, HCC1143, and HCC1187 were purchased from the American Type Culture Collection (Manassas, VA, USA). HeLa cells were obtained from the Cell Resource Center for Biomedical Research, Institute of Development, Aging and Cancer Tohoku University (Sendai, Miyagi, Japan). HCT116 and HCT116 p53 knockout (KO) cells were kindly provided by Dr. Bert Vogelstein (Johns Hopkins University, Baltimore, MD, USA). MBA-MB-468, MDA-MB-231, and HeLa cells were cultured in Dulbecco’s Modified Eagle’s Medium (DMEM; Nissui, Tokyo, Japan) supplemented with 10% heat-inactivated fetal bovine serum (FBS; Nichirei, Tokyo, Japan) and 5 mM glutamine. HCC38, HCC1143, and HCC1187 cells were cultured in RPMI-1640 (Nissui) medium supplemented with 10% FBS and 1% sodium pyruvate. HCT116 and HCT116 p53 KO cells were cultured in McCoy’s 5A (Gibco, Grand Island, NY, USA) medium supplemented with 10% FBS. All cells were grown in humidified cell culture incubators with 5% CO_2_ and 95% air at 37 °C.

### 2.2. Antibodies and Materials

Antibodies were purchased as follows: BIM (C34C5), BCL-XL (54H6), CREB (48H2) and cleaved caspase-3 (5A1E) from Cell Signaling Technology (Danvers, MA, USA); NOXA (114C307.1) from Thermo Fisher Scientific (Waltham, MA, USA); ATF-3 (C-19) and MCL-1 (S-19) from Santa Cruz Biotechnology (Santa Cruz, CA, USA); KLF4 (56CT5.1.6) from Bioss, Inc. (Woburn, MA, USA); α-tubulin (DM1A) and β-actin (AC-74) from Sigma-Aldrich (Tokyo, Japan). APTO-253 was purchased from MedChemExpress, LLC (Monmouth Junction, NJ, USA). Q-VD-OPh, Doxorubicin, etoposide, SP600125, SB203580, PD184352, and U0126 were purchased from Calbiochem (San Diego, CA, USA). Hydroxychloroquine (HCQ), Necrostatin-1 and Ferrostatin-1 were purchased from Cayman Chemical (Ann Arbor, MI, USA).

### 2.3. Cell Viability Assay

Cells were seeded in 96-well plate (Greiner Bio-one, Frickenhausen, Germany) at 5000 cells per well in 100 μL of medium, then treated with reagents as indicated the Figure legends for 24 h. After treatment, cell counts were measured using Cell Counting Kit-8 (CCK-8: Dojindo Molecular Technologies, Inc., Kumamoto, Japan). Briefly, 10 μL of the CCK-8 reagent was added to each well, then the plate was incubated for 2 h at 37 °C. The optical density (OD) at 450 nm was measured using a SpectraMax 250 microplate reader (Molecular Devices, San Jose, CA, USA). Cell viability for each sample was represented as the percentage relative to the untreated control.

### 2.4. Immunoblotting Analyses

Whole cell lysates were prepared with CHAPS lysis buffer [25 mM HEPES (pH 7.4), 250 mM NaCl, 1% CHAPS (3-[(3-Cholamidopropyl) dimethylammonio]-1-propanesulfonate)] in the presence of protease inhibitor cocktail (Nacalai, Kyoto, Japan). For immunoblotting analyses, 20 μg of protein was loaded on an SDS-polyacrylamide gel, transferred to a PVDF membrane, and analyzed by immunoblotting, as described previously [26]. The immunoblotting bands were quantified using ImageJ software and protein levels were normalized to the loading control b-actin or a-tubulin and fold change relative to the signal of loading control (set to 1).

### 2.5. RNA Interference

The retrovirus-encoding hairpin short-hairpin RNA (shRNA) vectors for NOXA, PUMA, BIM, BID, and MULE were cloned into the pSuper puro vector (Oligoengine, Seattle, WA, USA). The target sequences were as follows: 5′-GGAAACGGAAGATGGAATA-3′ (sh-*NOXA*), 5′-CTACCTCCCTACAGACAGA-3′ (sh-*BIM*), 5′-GGGTCCTGTACAATCTCAT-3′ (sh-*PUMA*), 5′-GGGATGAGTGCATCACAAA-3′ (sh-*BID*), 5′-TGCCGCAATCCAGACATAT-3′ (sh-*MULE*), 5′- GCACCTCTGCCACCGGATG-3′ (sh-ATF3 #1), 5′-GCAGAAAGTTCAACTTCCA-3′ (sh-ATF3 #2), and 5′-GCAGCTCATGCAACATCAT-3′ (sh-CREB). Retroviral infection was performed as previously described [13]. The lentiviral shRNA sh*KLF4*-expressing constructs were cloned into the plko.1 vector (Addgene, Cambridge, MA, USA). The target sequences were as follows: 5′-CCAGCCAGAAAGCACTACAAT-3′ (sh-KLF4) and 5′-CCTAAGGTTAAGTCGCCCTCG-3′ (sh-control). Lentiviral infection was performed as previously described [27]. Infected cells were selected using 1 μg/mL puromycin (Sigma-Aldrich) for three days.

### 2.6. Quantitative Real-Time PCR (qPCR)

Total RNA was extracted using the NucleoSpin RNA kit (Macherey Nagel, Germany) following the manufacturer’s instructions. Double-strand cDNA was prepared from total RNA using oligonucleotide (dT), random primers and Superscript III (Invitrogen, Carlsbad, CA, USA [27]. qPCR analysis was performed as previously described [27]. The following probes were predesigned from Applied Biosystems (Foster City, CA, USA): (β-actin, Hs03023880_g1; NOXA, Hs00560402_m1; KLF4, Hs00358836_m1). Data were calculated as mRNA expression levels relative to β-actin according to the manufacturer’s protocol. Data are shown as means ± SE for each group (*n* = 3).

### 2.7. Promoter Assay

The NOXA promoter region sequence between 925 bp upstream and 153 bp downstream of the transcriptional start site (TSS) was cloned into the KpnI and BglII sites of the PGV-B2 vector (Toyo B-Net, Tokyo, Japan). Each deleted NOXA promoter construct, −925 (−925 to +157), −171 (−171 to +157), −66 (−66 to +157), ΔCRE (−59 to +157), −168 (−168 to +157), −158 (−158 to +157), −148 (−148 to +157), −138 (−138 to +157), −128 (−128 to +157), -118 (−118 to +157), −108 (−108 to +157), −98 (−98 to +157), −88 (−88 to +157), −78 (−78 to +157), −66 (−66 to +157), and −59 (−59 to +157), was prepared by PCR. Control Renilla luciferase construct pRL-SV40 (0.1 μg; Promega, Madison, WI, USA) and Firefly luciferase reporter construct (1 μg) were co-transfected into cells with GeneJuice (Novagen, Madison, WI, USA). Luciferase activity was measured using a Dual-Glo luciferase assay system (Promega).

### 2.8. In Silico Analysis of Gene Expression and Kaplan–Meier Plots

The Kaplan–Meier plotter tool (http://kmplot.com/analysis/index.php?p=service&amp; accessed on 7 November 2020) was used to perform Kaplan–Meier survival analysis for breast cancer patients based on mRNA expression levels. Patients were categorized into high and low groups by expression profiles of KLF4 (220266_s_at), with the best performing threshold as the cutoff. A scatter plot of the correlations between KLF4 and PMAIP1 and between KLF4 and BBC3 expression were analyzed by GEPIA (http://gepia.cancer-pku.cn/detail.php?clicktag=correlation###; accessed on 5 November 2020)) using The Cancer Genome Atlas (TCGA) datasets for tumor and normal tissues.

### 2.9. UV-C Irradiation

UV-C irradiation was performed using the Stratalinker UV Crosslinker (Stratagene Cloning Systems, La Jolla, CA, USA). Before UV-C irradiation, the cell culture medium was removed. Cells were irradiated with UV light, after which the culture medium was added immediately.

### 2.10. Statistical Analysis

Each data value represents the mean ± S.E.M. for three separate experiments. The significance (such as *p* < 0.05) of differences between the experimental variables was determined using Welch’s *t*-test.

## 3. Results

### 3.1. UV Light Induces p53-Independent Activation of NOXA Expression and Caspase-3

**Figure 1:**
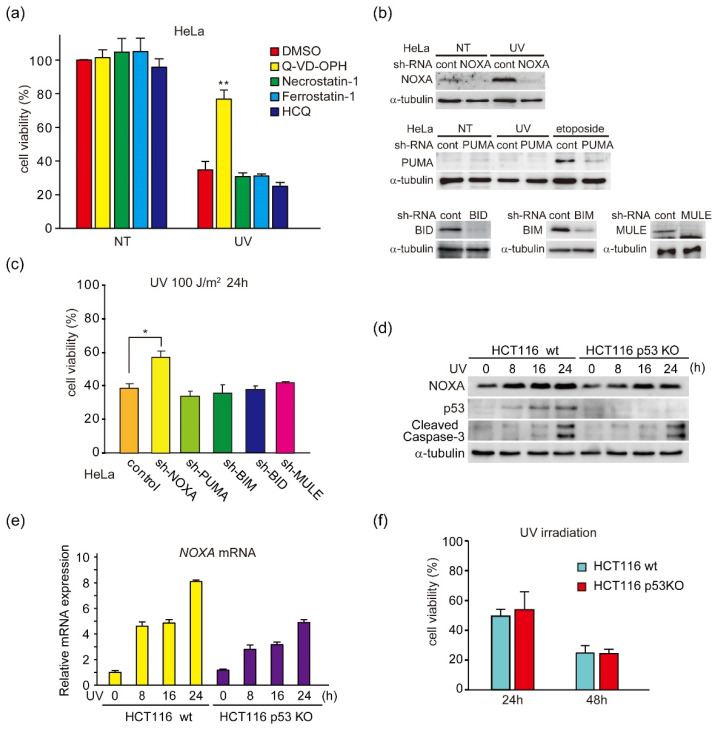
NOXA is a regulator of UV-induced apoptosis. (**a**) HeLa cells were irradiated with UV light (100 J/m^2^) in the absence (DMSO) or presence of programmed cell death inhibitor; apoptosis (Q-VD-OPH; 20 µM), necroptosis (Necrostatin-1; 30 µM), ferroptosis (Ferrostatin-1; 2 µM) and autophagy (HCQ; 20 µM). Error bars indicate S.E.M. (*n* = 3). (** *p* < 0.01, Welch’s *t*-test; *n* = 3). (**b**) HeLa cells stably expressing shRNA-targeting NOXA (sh-NOXA), PUMA (sh-PUMA), or sh-control were irradiated with UV light (100 J/m^2^) or stimulated by etoposide (100 µM). After a 24 h treatment, 20 μg of total cell lysates was subjected to immunoblot analysis with the indicated antibodies (upper panel). HeLa cells stably expressing shRNA-targeting BIM (sh-BIM), BID (sh-BID), MULE (sh-MULE), or sh-control were analyzed by immunoblot analysis (lower panel). Quantitative analysis of immunoblot results is shown Supplementary Figure S1a. (**c**) HeLa cells were irradiated with UV light (100 J/m^2^). After a 24 h treatment, cell viability was determined using CCK-8 analysis. Error bars indicate S.E.M. (*n* = 3). (* *p* < 0.05, Welch’s *t*-test; *n* = 3). (**d**–**f**) Cells were irradiated with UV light (100 J/m^2^). After the indicated treatment time, cells were analyzed by immunoblot analysis using indicated antibodies (**d**). Anti-cleaved caspase-3 antibody recognizes the large subunit of active caspase 3, immature p19 and fully mature p17 [34], but not the small subunit (p12) and uncleaved procaspase-3. In the following experiments, both p19 and p17 were often detected, but depending on the experimental condition, only active p17 was detected. Quantitative analysis of immunoblot results is shown in Supplementary Figure S1b. NOXA mRNA levels were determined by qPCR (**e**). Cell viability was determined using CCK-8 analysis. Error bars indicate S.E.M., (*n* = 3) (**f**).

In the present study, we found that the transcription factor KLF4 induces p53-independent apoptosis in TNBC cells via induction of NOXA by analysis of UV light-induced apoptosis in HeLa cells. Briefly, we first assessed the role of BH3-only proteins in the regulation of UV light-induced apoptosis, mediated by p53 [28], in HeLa cells that express wildtype p53. We found that UV light-induced cell death was mainly caused by apoptosis (Figure 1a). Cell death rates were reduced by Q-VD-OPH, an inhibitor of caspases that regulate and activate apoptosis. They were not affected by other inhibitors of programmed cell death commonly observed in cancer cells in response to stress, necroptosis, ferroptosis or autophagy [29]. The expression of BH3-only proteins, NOXA, PUMA, BIM, BID, and MULE, was suppressed by RNA interference-mediated gene knockdown (Figure 1b). Indeed, UV-induced expression of p53-inducible protein NOXA was not observed with NOXA knockdown (Figure 1b, upper). We previously found that NOXA-deficient mouse embryonic fibroblasts (MEFs) suppressed UV light-induced apoptosis more than p53-deficient MEFs [9]. This result suggests that p53-independent induction of NOXA by UV light can induce apoptosis. It has also been shown that UV light induces expression of another p53-inducible protein, PUMA [30]. However, under our experimental conditions, we did not detect induction of PUMA by UV light. It is possible that this was caused by the low amount of induced PUMA, which is supported by reports showing that fibroblasts and keratinocytes from NOXA knockout mouse are more resistant to UV light than those from PUMA knockout mouse [31]. Moreover, UV light-induced p53-independent induction of NOXA has also been observed in human cancer cells [32]. Our findings support this result. In contrast, the expression of PUMA was not observed, not even by UV light irradiation, but DNA-damaging agent etoposide (VP16)-induced induction was suppressed (Figure 1b, middle). As shown in Figure 1b,c, UV light-induced cell death, determined by a reduction in cell viability, was observed in HeLa cells and this cell death was suppressed by NOXA knockdown, but not by knockdown of PUMA, BIM, BID, or MULE. To analyze the role of p53 in UV light-induced NOXA expression, we analyzed NOXA expression in human colon cancer cell lines HCT116 with wild-type p53 (wt) and p53 knocked out (p53 KO) [33]. Induction of NOXA protein and mRNA by UV light exposure was weakly suppressed in p53 KO cells compared with p53 wt cells, which suggested that the suppression was dependent on p53 (Figure 1d,e). However, activating cleavage of caspase-3, which is the effector of apoptosis downstream of BAX and BAK, and induction of cell death were also observed in both wt and p53 KO cells (Figure 1d,f). These results suggest that NOXA is induced by UV light irradiation and stimulates apoptosis mainly through a p53-independent mechanism.

### 3.2. UV Light Induces NOXA Expression through the ERK Pathway

**Figure 2:**
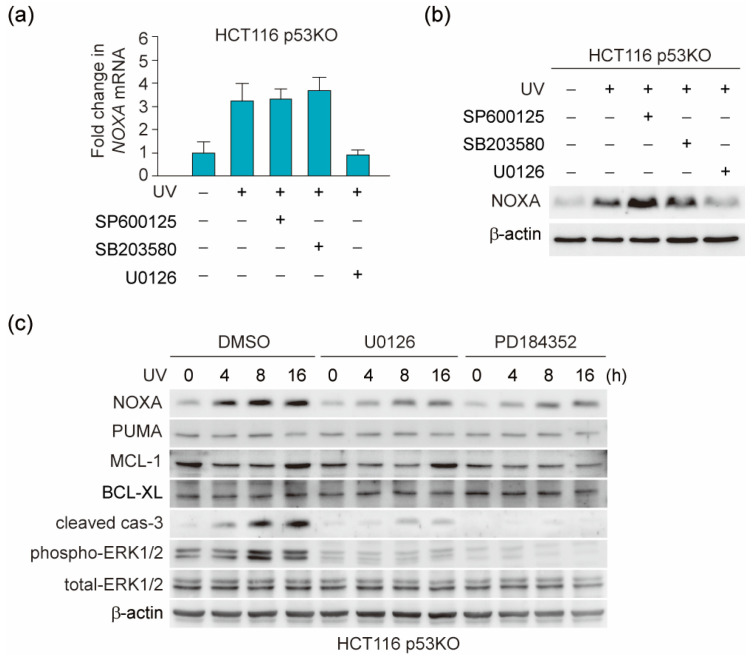
Extracellular signal-regulated kinase (ERK) activation induced by DNA-damage plays an important role in NOXA induction. (**a**,**b**) HCT116 p53 KO cells were treated for 30 min with 10 µM SP600125, 10 µM SB203580, or 20 µM U0126 prior to UV light (100 J/m^2^) irradiation. After 24 h of irradiation, NOXA mRNA expression levels were measured by qPCR (**a**), or 20 μg of total cell lysates was subjected to immunoblot analysis with the indicated antibodies (**b**). Quantitative analysis of immunoblot results is shown in Supplementary Figure S2a. (**c**) HCT116 p53 KO cells were treated for 30 min with 20 µM U0126 or 5 µM PD184352 prior to UV light (100 J/m^2^) irradiation. After the indicated irradiation time, cells were analyzed by immunoblot analysis with the indicated antibodies. Quantitative analysis of immunoblot results is shown in Supplementary Figure S2b.

Apoptosis is regulated by many intracellular signaling pathways, including the mitogen-activated protein kinase (MAPK) pathway [35]. UV signal has been shown to be regulated by c-Jun N-terminal protein kinase (JNK), because jnk1^−/−^/jnk2^−/−^ mice were resistant to UV light-induced apoptosis [36]. Therefore, we analyzed the effect of inhibitors of the MAPK family, which includes JNK, p38, and extracellular signal-regulated kinase (ERK) [37]. We used the following kinase inhibitors: 10 µM SP600125 (JNK inhibitor), 10 µM SB203580 (p38 inhibitor), and 20 µM U0126 (ERK inhibitor), which were previously reported to be effective in suppressing their respective kinase activity in HCT116 cells [38,39,40]. As shown in Figure 2a,b, the UV light-activated expression of NOXA mRNA and protein in HCT116 p53 KO cells was suppressed by ERK inhibitor U0126, but not by JNK inhibitor SP600125 or p38 inhibitor SB203580. This result was also supported by the previous findings showing that DNA-damage induced ERK dependent, but p53 independent, NOXA expression in both wild-type p53 and p53 null cancer cells [41]. To further clarify the involvement of ERK, the expression levels of NOXA and apoptosis regulators were analyzed following treatment with U0126 or another ERK inhibitor, PD184352 (Figure 2c). The expression of NOXA, cleavage of caspase-3, and activating phosphorylation of ERK1 and 2 were induced by UV light, while these effects were suppressed by both ERK inhibitors. In contrast, the expression of PUMA was not detected at the indicated time points. These results suggest that UV light induces ERK activation and this signaling pathway induces NOXA expression by a p53-independent mechanism. As PD184352 is effective for ERK1/2 at a lower concentration compared with U0126 (IC_50_ = 17 and 72 nM, respectively) [42,43], we performed the following experiments using PD184352.

### 3.3. Identification of UV-Inducible Element(s) in the NOXA Gene Promoter

**Figure 3:**
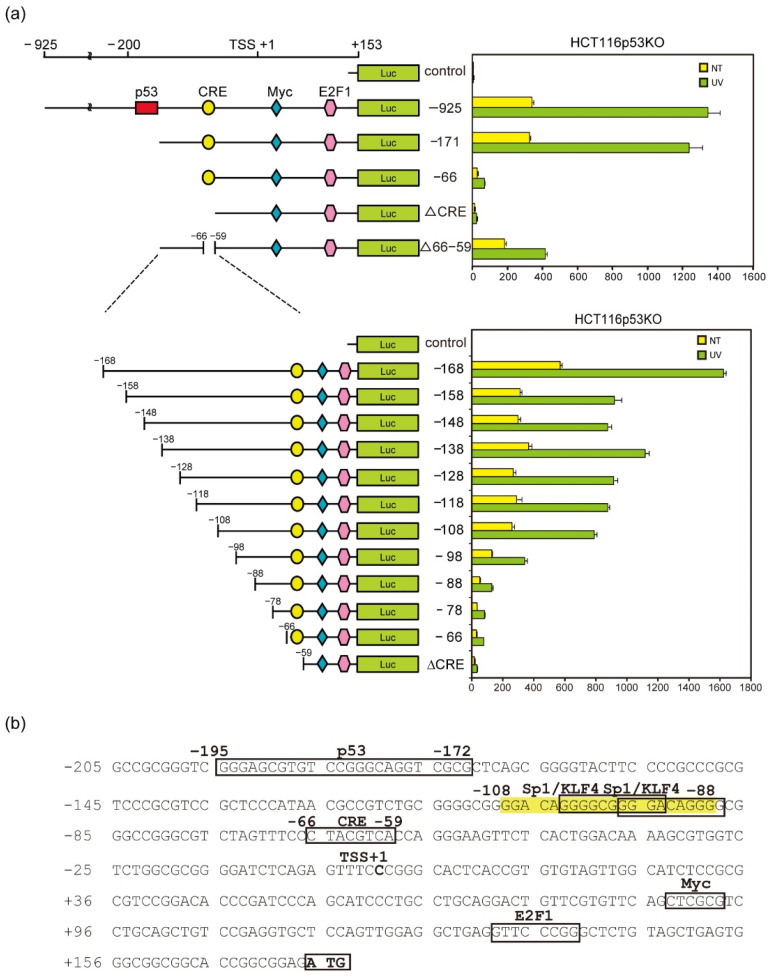
Identification of transcription factor binding sites in the *NOXA* promoter. (**a**) After 24 h of transfection of reporter constructs, cells were irradiated with UV light (green bars) or not subjected to irradiation (yellow bars) for 24 h, after which Firefly- and Renilla-dependent luciferase activities were determined. Firefly-dependent luciferase activity was normalized by the Renilla signals. Data are shown as the mean ± S.D. (*n* = 3). Each value represents the average of three independent experiments. (**b**) TFSEARCH analysis prediction of the transcription factor binding sites in the *NOXA* promoter region (−205/+153).

To determine the p53-independent induction mechanism of NOXA, we generated truncated human NOXA promoter luciferase constructs (Figure 3a). As previously reported, the NOXA promoter contains several functional transcription binding elements: p53 [8], activating transcription factor (ATF) 3/ATF4 [44], cyclic Adenosine monophosphate (AMP)-responsive element binding protein (CREB) [45], MYC [46], and adenovirus E2 promoter-binding factor 1 (E2F1) [47]. In HCT116 p53 KO cells, UV light-stimulated expression, as well as basal level expression, was highly induced in the NOXA promoter containing −925 to +153 from the transcription initiation site (−925 Luc) and in the −172 Luc lacking the p53 binding site (Figure 3a, upper). In −158 Luc, the promoter activity was decreased to about half of that in −168 Luc, but still showed high promoter activity. However, in −88 Luc, basal and UV light-induced promoter activities were decreased to approximately 6% of that in −168 Luc (Figure 3a, lower panel), which suggested that a UV light-responsive element(s) existed within this region. Additionally, transcription factors CREB and ATF3, which are thought to bind to and activate cAMP-response elements (CREs), are reportedly activated by UV light [48,49]. However, −66 Luc containing a CRE showed a weak basal level of promoter activity and weak induction by UV light. These results support the results showing that CRE is not the main UV-responsive element of the NOXA promoter (Figure 3a). Furthermore, −98 Luc showed a decrease in promoter activity compared with −108 Luc. This result suggested that a DNA element important for basal and UV light-induced promoter activity existed between −108 and −66, especially in the region containing −98. Therefore, we searched for putative transcription factor binding sites in this region via TFSEARCH database analysis (http://www.cbrc.jp/research/db/TFSEARCH.html; accessed on 20 October 2011), and found that two overlapping Sp1/KLF4 binding motifs exist in the −108 to −88 region (Figure 3b).

### 3.4. KLF4 Knockdown Inhibits the Induction of NOXA and Activation of Caspase-3 by UV Light

**Figure 4:**
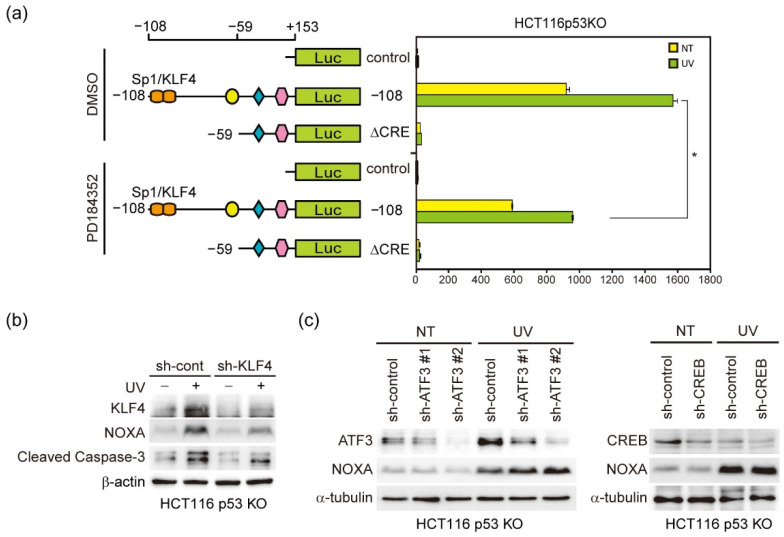
KLF4 is responsible for the p53-independent induction of NOXA expression by UV light irradiation. (**a**) After 24 h of transfection of reporter constructs, HCT116 p53 KO cells were treated with PD184352 (5 µM) prior to UV light irradiation. Cells were irradiated with (green bars) or without (yellow bars) UV light for 24 h, after which Firefly- and Renilla-dependent luciferase activities were determined. Firefly-dependent luciferase activity was normalized by the respective Renilla signal. Data are shown as the mean ± S.D. (*n* = 3). Each value represents an average from three independent experiments. (**b**,**c**) HCT116 p53 KO cells stably expressing shRNA-targeting KLF4 (sh-KLF4), ATF3 (sh-ATF3 #1, sh-ATF3 #2), CREB (sh-CREB) or sh-control were irradiated with UV light (100 J/m^2^). After 24 h of irradiation, 20 μg of total cell lysates was subjected to immunoblot analysis with the indicated antibodies. Quantitative analysis of immunoblot results is shown in Supplementary Figure S3a,b.

The above results suggest that Sp1/KLF4 elements are involved in the induction of NOXA expression by UV light. Therefore, we then investigated whether these elements were regulated by ERK activity, as shown in Figure 2a. Both basal level and UV light-induced activity of the *NOXA* promoter were suppressed by ERK inhibitor PD184352 (Figure 4a). We further analyzed the role of KLF4 in UV light-induced expression of NOXA and apoptosis in HCT116 p53 KO cells. As expected, KLF4 knockdown (Figure 4b) partially inhibited both the expression of NOXA and activation of caspase-3 (Figure 4c). In addition, although the rate of suppression is not high, knockdown of UV light-inducible transcription factors ATF3 and CREB that potentially bind to CRE did not significantly affect the expression of NOXA (Figure 4c). These results supported the results showing that the CRE is not the main UV-responsive element of the NOXA promoter (Figure 3a). It has been shown that ATF3 activated p53-independent NOXA induction by cisplatin [44], suggesting that the involvement of KLF4 and ATF3 in DNA damage varies by cell type or other contexts.

### 3.5. Involvement of KLF4 in the Expression of NOXA in Cancer Cells and in Survival of Breast Cancer Patients

**Figure 5:**
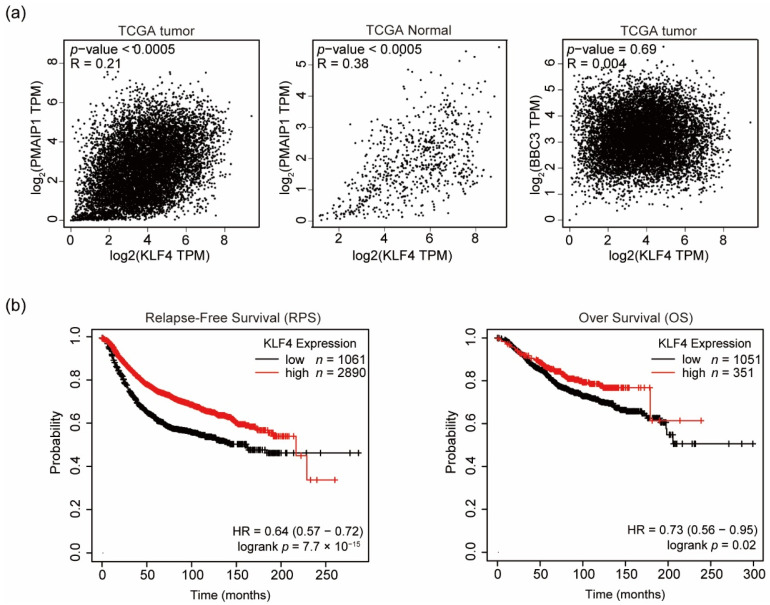
Correlation between KLF4 and NOXA expression in breast cancer tissues and the effect of KLF4 expression in breast cancer patients. (**a**) The correlation between KLF4 expression and NOXA (gene name PMAIP1; left and middle) and PUMA (gene name BBC3; right) expression based on the Cancer Genome Atlas (TCGA) dataset are illustrated. (**b**) Kaplan–Meier curves of relapse-free survival (RFS; left panel) and overall survival (OS; right panel) in breast cancer patients were plotted according to the KLF4 expression levels (high vs. low). The *p*-value of the log-rank test, hazard ratio, and confidence interval are shown in each figure.

Next, we analyzed whether KLF4 is involved in NOXA expression in human cancers and normal tissues. We found a weak but significant positive correlation between KLF4 and NOXA (gene name PMAIP1) mRNA expression levels (R-score = 0.21 in cancers, R-score = 0.38 in normal tissues) using the total dataset of Gene Expression Profiling Interactive Analysis database (Figure 5a). In contrast, we investigated the expression of PUMA, another inducer of p53, as a control. We found no significant correlation between KLF4 and PUMA (gene name BBC3) mRNA expression levels (R-score = 0.004). These results support the possibility that the expression of NOXA is regulated by KLF4.

KLF4 has been shown to function as a tumor-promoting factor in many cancers [50,51]. However, KLF4 can also act as a tumor suppressor in a context-dependent manner [50]. As KLF4 induces expression of proapoptotic factor NOXA in a p53-independent manner, we considered analysis of cancers in which KLF4 acts as a tumor suppressor in a p53-independent manner. About 80% of TNBC tumors contain p53 mutations and TNBC patients have a poor prognosis [19,52]. Considering the function of NOXA, the only target of NOXA has been determined to be the antiapoptotic factor MCL1. MCL1 knockdown induces apoptosis in a subset of TNBC cell lines [53]. These findings suggest that TNBC cell survival and death are at least partially regulated by the balance between NOXA and MCL1. However, because we did not assess the expression levels of KLF4 or the probability of survival in TNBC cells, we next analyzed the role of KLF4 and NOXA in overall breast cancers without classification of hormone receptor expression levels or the p53 mutation status. We investigated the potential correlation between KLF4 expression and relapse-free survival (RFS) and overall survival (OS) rates of breast cancer patients using the Kaplan–Meier plotter database. As shown in Figure 5b, breast cancer patients with high levels of KLF4 expression had relatively good RFS and OS rates. These results suggest that KLF4 functions as a tumor suppressor in some, if not all, breast cancers.

### 3.6. UV-Light and Doxorubicin Induce ERK-Mediated NOXA Induction and Apoptosis in TNBC Cell Lines

**Figure 6:**
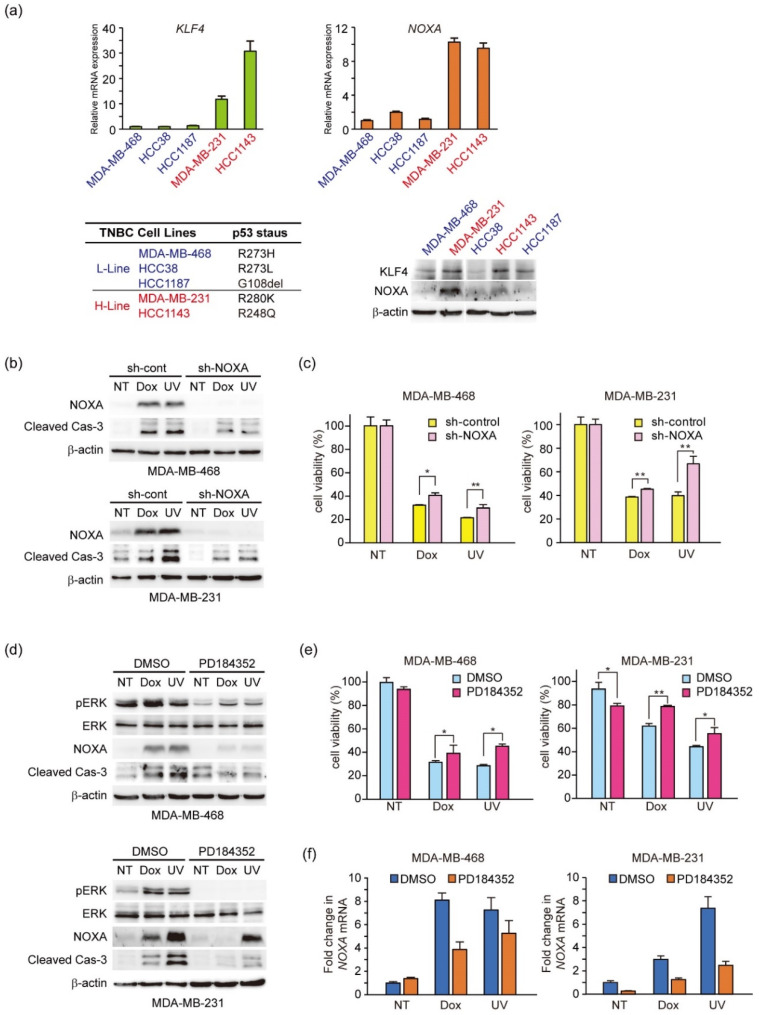
ERK activation and induction of NOXA by DNA damage-induced apoptosis in triple-negative breast cancer (TNBC) cells. (**a**) KLF4 (left, upper panel) and NOXA (right, upper panel) mRNA levels in the indicated cell lines were measured by qPCR. The mutation status of the p53 gene in TNBC cell lines is shown (left, lower panel). Total cell lysates (20 μg) were subjected to immunoblot analysis with the indicated antibodies (right, lower panel). Quantitative analysis of immunoblot results is shown in Supplementary Figure S4a. (**b**,**c**) TNBC cell lines (MDA-MB-468 and MDA-MB-231) that stably expressed shRNA-targeting NOXA (sh-NOXA) or sh-control were treated with doxorubicin (1 μg/mL) or irradiated with UV light (100 J/m2). After 24 h of treatment, 20 μg total cell lysates was subjected to immunoblot analysis with the indicated antibodies (**b**) and cell viability was measured by CCK-8 assays (**c**). Quantitative analysis of immunoblot results is shown in Supplementary Figure S4b. Error bars indicate S.E.M. (*n* = 3). (* *p* < 0.05, ** *p* < 0.01, Welch’s t-test; *n* = 3) (**d**–**f**) TNBC cell lines (MDA-MB-468 and MDA-MB-231) were treated for 30 min with 5 µM PD184352 prior to doxorubicin (1 μg/mL) treatment or UV light (100 J/m^2^) irradiation. After 24 h, the cells were analyzed by immunoblotting with the indicated antibodies (**d**), cell viability was measured by CCK-8 assays (**e**) and NOXA mRNA levels were measured by qPCR (**f**). Quantitative analysis of immunoblot results is shown in Supplementary Figure S4c. Error bars indicate S.E.M. (*n* = 3). (* *p* < 0.05, ** *p* < 0.01, Welch’s *t*-test; *n* = 3).

To investigate the role of NOXA in the apoptosis of TNBCs, we analyzed five TNBC cell lines with a mutated p53 gene and found cells in which both KLF4 and NOXA mRNA and protein expression levels were high (H-lines MDA-MB-231 and HCC1143) or low (L-lines MDA-MB-468, HCC38, and HCC1187) (Figure 6a). We further assessed whether UV light induced ERK-mediated expression of NOXA and NOXA-mediated apoptosis in L-line MDA-MB-468 and H-line MDA-MB-231 cells as observed in HeLa and HCT116 cells. In this experiment, the effect of DNA-damaging agent doxorubicin, which is also used for treatment of TNBC [50], was analyzed simultaneously. As a result, UV light and doxorubicin induced NOXA protein expression and caspase-3 activation in both cell types (Figure 6b). Moreover, NOXA knockdown partially reduced apoptosis induction (Figure 6c) by these stimuli that induce DNA damage, which suggested that DNA damage induced NOXA-mediated apoptosis in TNBC cells. Additionally, ERK inhibitor PD184352 suppressed DNA damage-induced NOXA protein expression, Caspase-3 activation, and apoptosis in both types of cells (Figure 6d–f). In MD-MB-468 cells, expression of NOXA protein and mRNA induced by Dox and UV light was suppressed by PD184352 (Figure 6d, upper and Figure 6f, left). Conversely, in MDA-MB-231 cells, the degree of NOXA protein suppression by UV light was not as high (Figure 6d, lower). As the amount of NOXA protein is regulated by the ubiquitin-proteasome system [51], it is possible that UV light-, but not Dox-, specific protein stabilization signals operated specifically in MDA-MB-231 cells. In any case, these results suggest involvement of DNA damage-induced NOXA expression mediated by the ERK pathway in apoptosis of p53-mutated TNBC cells, such as p53-independent apoptosis.

### 3.7. KLF4 Induces Both the Expression of NOXA and Cell Death in TNBC Cell Lines

**Figure 7:**
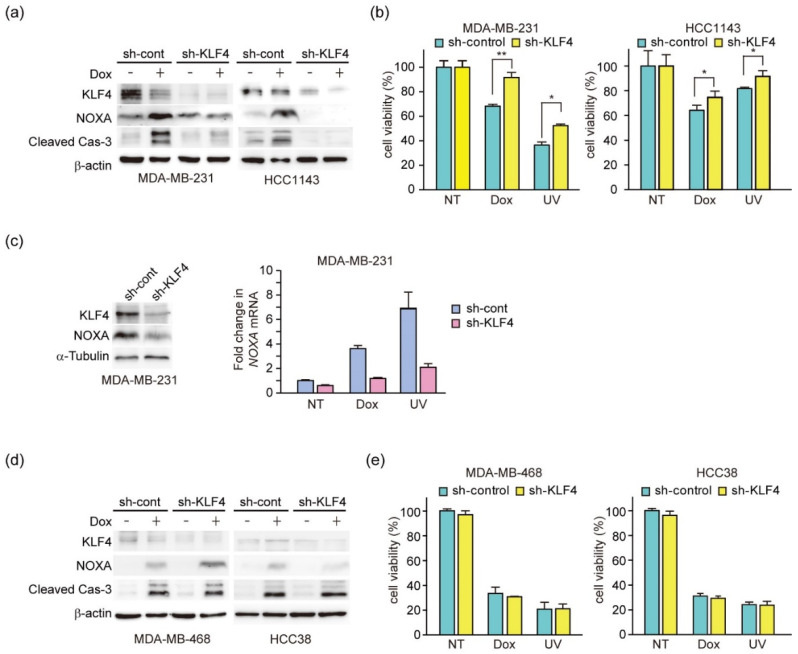
Anti-cancer drug-induced DNA damage elicits NOXA-dependent apoptosis enhanced by high expression of KLF4. (**a**,**b**) TNBC cell lines (MDA-MB-231 and HCC1143) that stably expressed shRNA-targeting KLF4 (sh-KLF4) or sh-control were treated with doxorubicin (1 μg/mL) or irradiated with UV light (100 J/m^2^). After 24 h of treatment, 20 μg total cell lysates was subjected to immunoblot analysis with the indicated antibodies (**a**) and cell viability was measured by CCK-8 assays (**b**). Quantitative analysis of immunoblot results is shown in Supplementary Figure S5a. Error bars indicate S.E.M. (*n* = 3). (* *p* < 0.05, ** *p* < 0.01, Welch’s t-test; *n* = 3). (**c**) MDA-MB-231 cells that stably expressed shRNA-targeting KLF4 (sh-KLF4) or sh-control were subjected to immunoblot analysis with the indicated antibodies (left). Cells were treated with doxorubicin (1 μg/mL) or irradiated with UV light (100 J/m^2^). After 24 h, NOXA mRNA expression levels were measured by qPCR (right). Quantitative analysis of immunoblot results is shown in Supplementary Figure S5b. (**d**,**e**) TNBC cell lines (MDA-MB-468 and HCC38) that stably expressed shRNA-targeting KLF4 (sh-KLF4) or sh-control were treated with doxorubicin (1 μg/mL) or irradiated with UV light (100 J/m^2^). After 24 h of treatment, 20 μg total cell lysates were subjected to immunoblot analysis with the indicated antibodies (**d**) and cell viability was measured by CCK-8 assays (**e**). Quantitative analysis of immunoblot results is shown in Supplementary Figure S5c.

Next, we analyzed the role of KLF4 in DNA damage-induced apoptosis in H- and L-lines. First, induction of NOXA protein, Caspase-3 activation, and apoptosis by doxorubicin in H-lines MDA-MB-231 and HCC1143 was suppressed by KLF4 knockdown (Figure 7a,b), which suggested that KLF4-induced NOXA was involved in DNA-damage induced apoptosis in H-lines. Unexpectedly, Dox reduced KLF4 expression in MDA-MB-231 cells, but not in HCC1143 cells. However, KLF4 knockdown suppressed NOXA expression and induction of apoptosis (Figure 7a,b). It has been reported that the transcriptional activity of KLF4 is regulated post-transcriptionally [52]. Therefore, it is possible that Dox enhanced the transcriptional activation ability of KLF4 to induce NOXA, although Dox reduced the amount of KLF4 protein in MDA-MB-231 cells. We also found that the induction of NOXA mRNA was suppressed by KLF4 knockdown in MDA-MB-231 cells (Figure 7c), which supported that KLF4 activated transcription of the *NOXA* gene. Conversely, in L-line MDAMB-468 and HCC38 cells, the expression level of KLF4 protein was low and KLF4 knockdown did not affect the induction of NOXA protein, Caspase-3 activation, or apoptosis by doxorubicin (Figure 7d,e). These results suggest that NOXA-mediated mechanism other than KLF4 in L-lines partially induces apoptosis. Indeed, NOXA knockdown attenuated apoptosis in L-Line MDA-MB-468 cells (Figure 6c).

### 3.8. KLF4-Activating Agent APTO-253 Induces Cell Death in TNBC Cells

**Figure 8:**
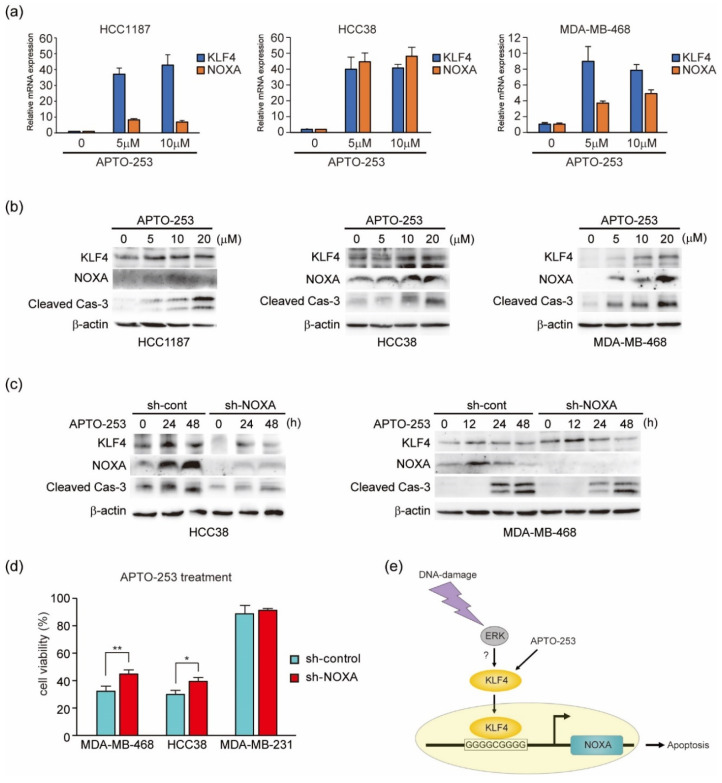
APTO-253 promotes KLF4 mRNA expression and induces NOXA-mediated apoptosis in TNBC cell lines with low KLF4 expression. (**a**,**b**) Cells were exposed to the indicated amounts of APTO-253 (5–20 µM). After 24 h of treatment, the indicated mRNA expression levels in MBA-MB-468 (**left**), HCC38 (**middle**) and HCC1187 (**right**) cells were measured by qPCR (**a**), and 20 μg of total cell lysates was subjected to immunoblot analysis with the indicated antibodies (**b**). Quantitative analysis of immunoblot results is shown in Supplementary Figure S6a. (**c**,**d**) HCC38, MDA-MB-468 or MDA-MB-231 cells stably expressing shRNA-targeting NOXA (sh-NOXA) or sh-control were treated with APTO-253 (5 µM). After the indicated treatment time, MDA-MB-231 and HCC38 cells were analyzed by immunoblot analysis (**c**). After 24 h of treatment, cell viability was determined by CCK-8 analysis (**d**). Error bars indicate S.E.M. (*n* = 3). (* *p* < 0.05, ** *p* < 0.01, Welch’s t test; *n* = 3). Quantitative analysis of immunoblot results is shown in Supplementary Figure S6b. (**e**) Schematic of KLF4-mediated induction of NOXA by DNA-damage or APTO-253.

The above results indicate that NOXA is partially involved in the induction of apoptosis in both TNBC L-lines and H-lines. As it has been reported that apoptosis-inducing function of NOXA is suppressed in constitutive NOXA-high expressing cancer cells—especially in hematopoietic cancers [53]—we considered that increasing the induction of NOXA by KLF4 in L-lines enhances the induction of apoptosis. Therefore, we analyzed the effect of KLF4-activating agent APTO-253 in L-lines, which may correspond to KLF4-low expression patients (Figure 5b). As previously shown [24,25], APTO-253 activated mRNA expression of KLF4 in L-lines (Figure 8a). Moreover, mRNA expression of NOXA was also induced by APTO-253. Protein expression of KLF4 and NOXA, as well as activation of caspase-3, was induced by APTO-253 in a dose-dependent manner (Figure 8b). Although NOXA mRNA and protein expression levels differed among these cell lines after APTO-253 treatment, it is possible that these differences were caused by the histone modification status around the NOXA gene as well as protein modification and translation efficiency in each cell line. As APTO-253 has also been shown to suppress the expression of MYC [25], we analyzed whether APTO-253 induces apoptosis through targets other than KLF4. As shown in Figure 8c, APTO-253-induced caspase-3 activation was partially suppressed by NOXA knockdown. Furthermore, APTO-253-induced cell death was also partially suppressed by NOXA knockdown (Figure 8d). In contrast to these results, as expected, APTO-253 did not enhance apoptosis in H-line MDA-MB-231 cells. These results suggest that a DNA-damage-evoked ERK signal activates KLF4, and that activated KLF4 induces transcriptional activation of NOXA. Although it is possible that APTO-253 acts on other apoptosis regulators, our results indicate that APTO-253 induces NOXA-mediated apoptosis in TNBC L-line cells (Figure 8e). Our findings suggest that APTO-253 may be a candidate therapeutic agent for TNBC.

## 4. Discussion and Conclusions

In the present study, we showed that induction of ERK and KLF4, which leads to transcriptional activation of NOXA, is involved in p53-independent apoptosis in response to DNA damage stressors such as UV light. Firstly, we analyzed UV light-induced apoptosis in HeLa and HCT116 cells, in which this phenomenon has already been analyzed [54], and found that p53-independent induction of NOXA is involved in UV light-induced apoptosis. Next, we showed that p53-independent induction of NOXA by UV light was regulated by ERK signaling in HCT116 p53 KO cells. Moreover, we found that the region containing potential KLF4-and CRE-binding sites in the NOXA promoter was responsive to UV light and ERK signaling, and that KLF4, but not CRE-binding factors ATF3 and CREB, induced the expression of NOXA in response to UV light (Figure 4c). However, the induction of apoptosis by UV light is difficult to apply as a cancer treatment, so we proceeded with analyzing cancers in which the KLF4-NOXA axis is functionally active. We chose TNBCs, which frequently have p53 mutations. TNBC cell lines were divided into high and low groups based on their expression levels of both KLF4 and NOXA. The anticancer drug doxorubicin, which causes DNA damage, also significantly induced KLF4-NOXA-mediated apoptosis in the high TNBC cell line group. Moreover, in low expression cell lines, we also found that the KLF4 activator APTO-253 induced activation of NOXA expression and apoptosis. DNA damage-induced p53-independent apoptosis has been thought to be mainly caused by Forkhead box O (FOXO) transcription factors, which induce BIM or p73, or c-MYC that induces PUMA [55,56]. Our study revealed a novel mechanism by which the KLF4-NOXA axis is involved in the induction of p53-independent apoptosis in response to DNA damage. Furthermore, although the involvement of other apoptosis-inducing factors beyond NOXA cannot be ruled out, our results suggest that activation of the KLF4-NOXA pathway can be applied to the treatment of TNBCs.

Cancer cells can develop a variety of strategies to avoid apoptosis for survival. To do this, they often use dysregulation of BCL-2 family apoptosis regulators [1,2]. Indeed, amplification of the antiapoptotic genes *BCLX* and *MCL1* were detected by high resolution analyses of somatic copy number alterations in a significant number of human cancers [57]. The most common cell survival strategy is p53 loss-of-function. Under normal conditions, p53 helps eliminate cells that have oncogene-driven dysregulated cell cycle control [5], as well as cells with other apoptosis-inducing signals evoked [2]. Studies in which researchers have knocked out certain genes, including NOXA, PUMA, BAX and BAK, have revealed that the BCL-2 family-regulated apoptosis-inducing pathway is a critical target for p53 and DNA damage that is commonly evoked by cytotoxic anticancer agents [7,9,58]. Therefore, it is important to activate this pathway to induce apoptosis in cancer cells that have a mutated version of p53. Moreover, it has been demonstrated that the ERK-dependent pathway to NOXA expression regulates apoptosis by DNA-damage; however, the underlying mechanism has still not been elucidated [41]. Our present study demonstrates that KLF4 is a transcriptional activator of NOXA and that the KLF4 activator APTO-253 induces apoptosis in p53-mutated TNBC cells via the induction of NOXA expression. At present, several reports have described the relationship between KLF4 and NOXA. For example, it has been shown that overexpression of KLF4 in neuroblastoma cells induced apoptosis and expression of the pro-apoptotic proteins BAX, NOXA, PUMA and p53, while it also decreased expression of the anti-apoptotic proteins BCL-2 and MCL-1 [59]. Moreover, it has also been demonstrated that inhibition of KLF4 expression in melanoma cells decreased mRNA expression levels of BCL-2 and BCL-XL, while increasing expression of the pro-apoptotic factors NOXA, BAX and p53 [60]. In contrast, our present study identified a potential KLF4 binding element in the NOXA promoter, and showed a strong correlation between KLF4 expression, NOXA induction and apoptosis in TNBC cells. Moreover, our results suggest a novel mechanism underlying UV light- and DNA damage-induced apoptosis.

It has been suggested that UV irradiation leads to the clustering of cell surface receptors, including epidermal growth factor receptor (EGFR), and the generation of reactive oxygen species that directly activate EGFR. This results in autophosphorylation and activation of intracellular signaling pathways downstream of EGFR [61]. Such cell surface receptors can transduce signals via the RAS-MAPK pathway, which activates JNK, p38, and ERK [62]. Among MAPKs, the proapoptotic role of stress-activated kinases JNK and p38 are well documented [63]. For example, JNK knockout mice were resistant to UV light-induced apoptosis [36]. In contrast, ERK signaling is known to inhibit mitochondria-mediated apoptosis through activation of BH3-only proteins, such as BIM and BIK, and also inhibit degradation of the antiapoptotic protein MCL-1 by direct phosphorylation [62]. However, ERK activity is involved in cell death induced by various anticancer agents [64]. Moreover, it has been shown that MEK inhibitors can attenuate UV light-induced apoptosis [65], which suggests a role for ERK in this process. Additionally, KLF4 nuclear export requires phosphorylation at S132 via ERK activation [66], but a specific role of the ERK-KLF4 pathway in UV light-induced apoptosis was still not elucidated. Therefore, our results suggest a novel mechanism of UV light-induced apoptosis in addition to the JNK pathway. Further analysis of this pathway will provide a greater understanding of the mechanism underlying UV light-induced skin carcinogenesis and help with the development of a novel method to treat it.

KLF4 is a transcription factor that reprograms somatic cells into pluripotent stem cells in cooperation with octamer-binding transcription factor 4 (OCT4), sex-determining region Y-box 2 (SOX2), and MYC [67]. KLF4 has context-dependent oncogenic or tumor-suppressor functions. Moreover, KLF4 has been shown to function as a tumor-promoting factor in cancers, and also act as a reprograming factor to generate and maintain cancer stem cells [68,69]. KLF4 was also determined to be a gene that bypasses oncogenic RAS-induced senescence by suppressing the expression of p53, but also inhibits cell proliferation in normal fibroblasts by inducing the cell-cycle inhibitor p21 [70]. Several reports have shown that KLF4 can induce apoptosis in different types of cancer cells [52]. For example, overexpression of KLF4 promoted apoptosis through activation of BAX expression in breast cancer cells [71]. Therefore, it is believed that the tumor-promoting and suppressor functions of KLF4 are regulated in a context-dependent manner [72]. A proapoptotic function of KLF4 has been reported in breast cancer cells [71], and antitumor activity of KLF4-inducer, APTO-253, was also observed in patients with advanced or metastatic solid tumors [73]. However, the underlying precise mechanism of KLF4-induced apoptosis was not fully elucidated in these reports. In this study, our findings suggest that NOXA is transcriptionally activated by KLF4, which can then induce apoptosis in UV light and DNA-damaging agent-stimulated cells and TNBC cells. Therefore, following analysis of the role of the KLF4-NOXA pathway in the treatment of various cancer types, we believe that the use of APTO-253 itself, or in combination with other anticancer drugs, should be examined for cancer treatment.

TNBCs represent about 16% of total breast cancer cases [74]. They occur frequently in young women and tend to exhibit aggressive, metastatic behavior [75]. As therapeutic molecular targets found in other type of breast cancers, such as ER, PR, and HER2, are not expressed in TNBCs, these patients tend to have poorer prognoses, caused by their high metastatic progression [19]. Currently, cytotoxic agents such as microtubule stabilizers (such as taxanes), anthracyclines (doxorubicin and epirubicin), and platinum agents (carboplatin and cisplatin) are used to treat TNBCs [19,76]. However, because TNBC tumors have a high rate of p53 mutations, the effects of these drugs may be limited. Therefore, we believe that further detailed analysis of the roles and activation mechanisms of KLF4 and NOXA will help to clarify a relevant molecular mechanism that can be used in the development of novel treatment methods for TNBC and other p53 mutant cancer cells.

## Data Availability

The data presented in this study are available in the article and supplementary materials.

## Supplementary Materials

The following are available online at https://www.mdpi.com/article/10.3390/genes12040539/s1, Figure S1: Densitometric analysis of relative protein level as shown in Figure 1; Figure S2: Densitometric analysis of relative protein level as shown in Figure 2; Figure S3: Densitometric analysis of relative protein level as shown in Figure 4; Figure S4: Densitometric analysis of relative protein level as shown in Figure 6; Figure S5: Densitometric analysis of relative protein level as shown in Figure 6; Figure S6: Densitometric analysis of relative protein level as shown in Figure 8.
