# Supplementary material for: Krüppel-Like Factor 4 and Its Activator APTO-253 Induce NOXA-Mediated, p53-Independent Apoptosis in Triple-Negative Breast Cancer Cells
Source: Genes (Basel). 2021 Apr 8;12(4):539. doi: 10.3390/genes12040539 (PMC8068402; doi:10.3390/genes12040539)
Supplement: Supplementary file 1 [file genes-12-00539-s001.pdf]

Supplementary Information for

Krüppel-like factor 4 and its activator APTO-253 induce NOXA-mediated, p53-independent apoptosis in triple-negative breast cancer cells

Wataru Nakajima, Kai Miyazaki, Yumi Asano, Satoshi Kubota, and Nobuyuki Tanaka*

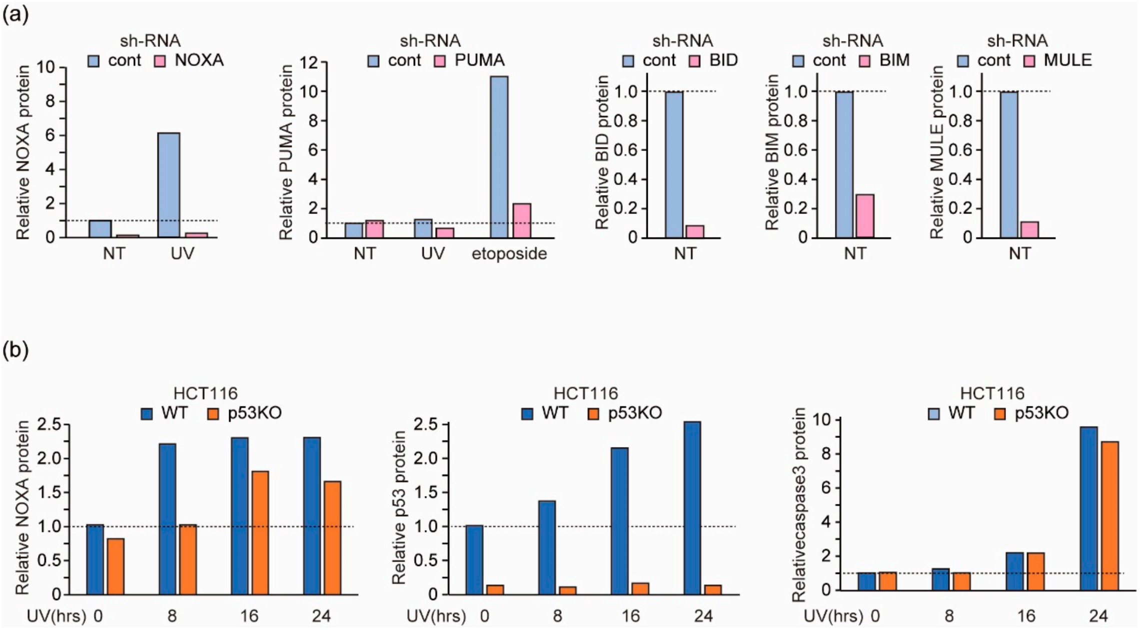

Figure S1. Densitometric analysis of relative protein level as shown in Figure 1. Indicated protein levels were normalized to total loading control protein. **a**, Densitometric analysis of relative protein level as shown in Figure 1b. **b**, Densitometric analysis of relative protein level as shown in Figure 1d.

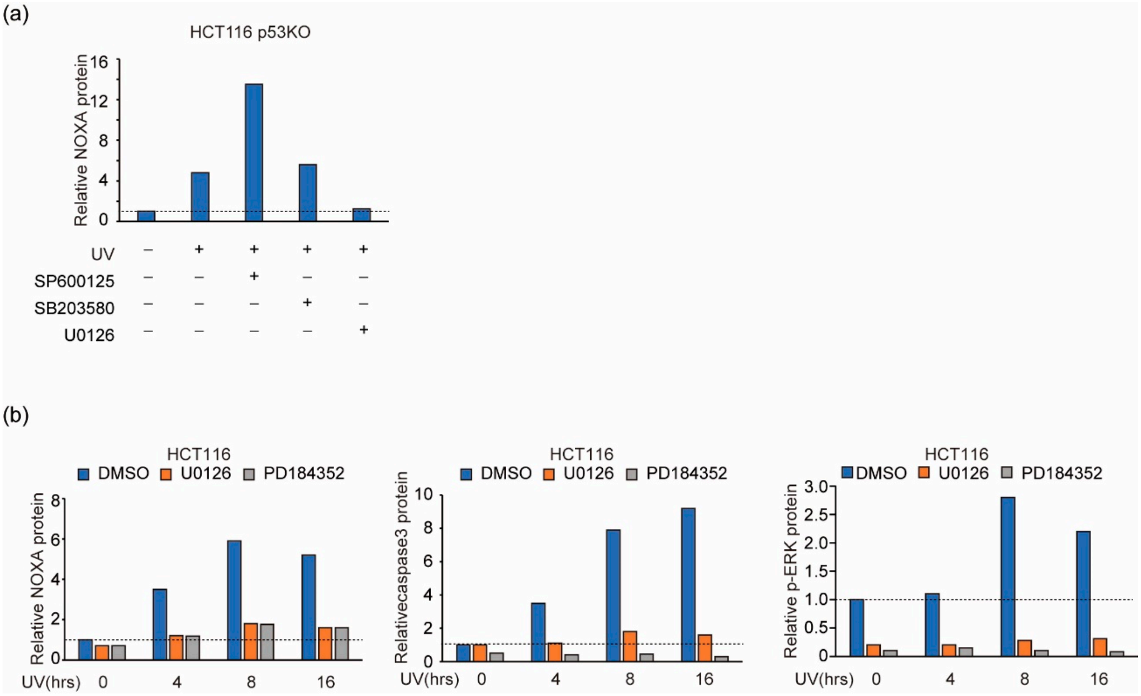

Figure S2. Densitometric analysis of relative protein level as shown in Figure 2. Indicated protein levels were normalized to total loading control protein. **a,** Densitometric analysis of relative protein level as shown in Figure 2b. **b,** Densitometric analysis of relative protein level as shown in Figure 2c.

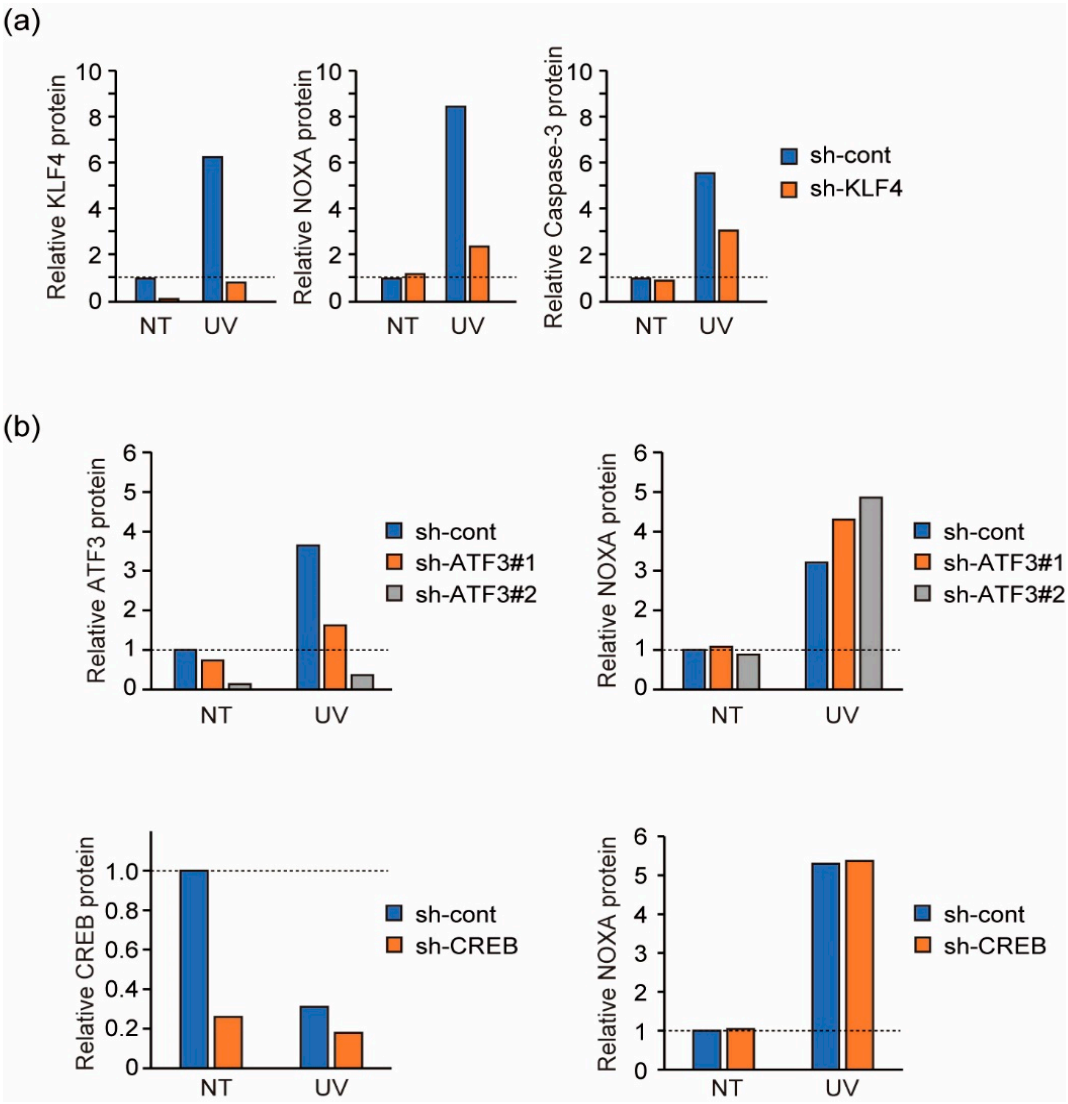

Figure S3. Densitometric analysis of relative protein level as shown in Figure 4. Indicated protein levels were normalized to total loading control protein. **a,** Densitometric analysis of relative protein level as shown in Figure 4b. **b,** Densitometric analysis of relative protein level as shown in Figure 4c.

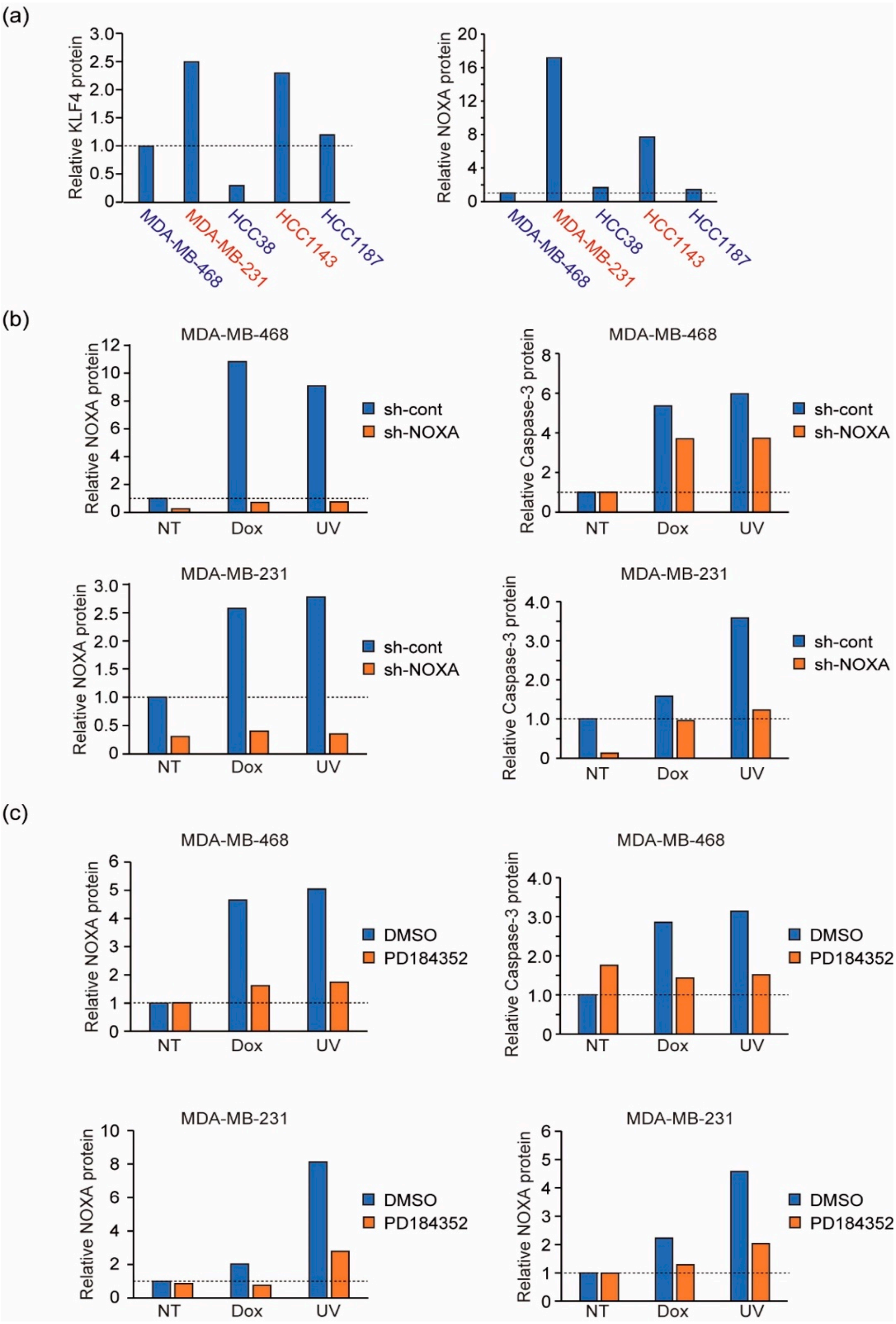

Figure S4. Densitometric analysis of relative protein level as shown in Figure 6. Indicated protein levels were normalized to total loading control protein. **a,** Densitometric analysis of relative protein level as shown in Figure 6a. **b,** Densitometric analysis of relative protein level as shown in Figure 6b. **c,** Densitometric analysis of relative protein level as shown in Figure 6d.

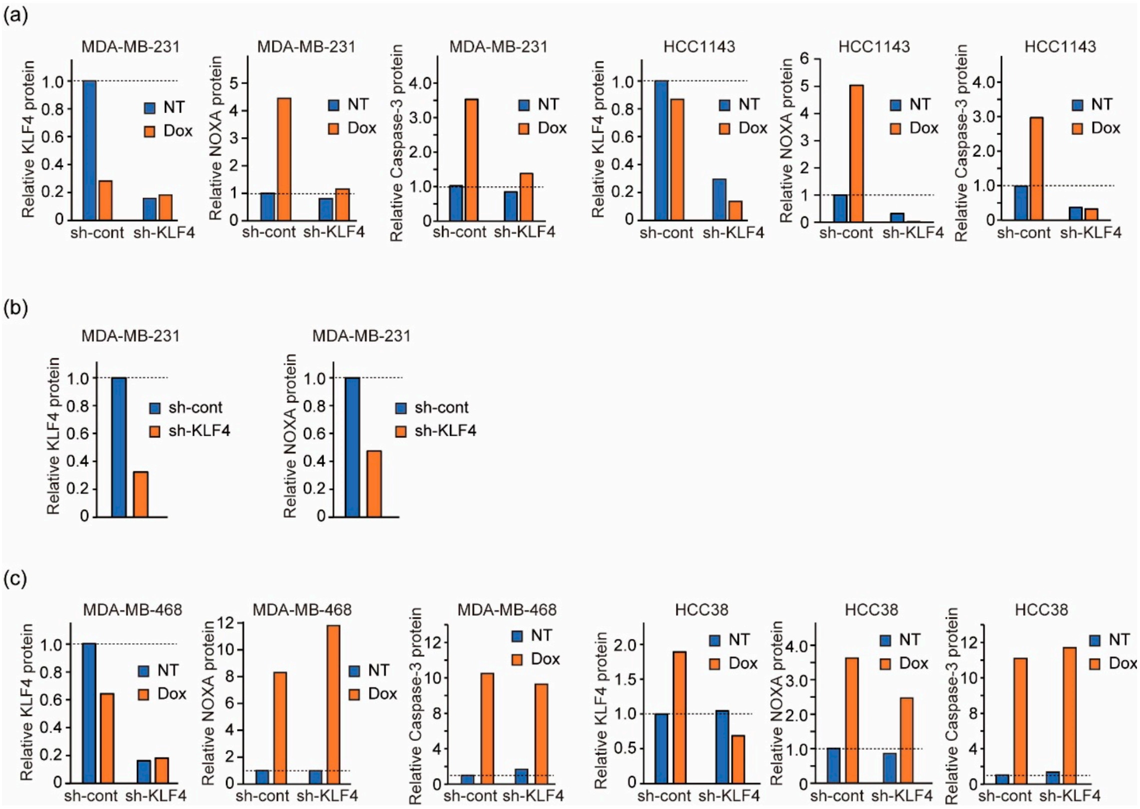

Figure S5. Densitometric analysis of relative protein level as shown in Figure 7. Indicated protein levels were normalized to total loading control protein. **a,** Densitometric analysis of relative protein level as shown in Figure 7a. **b,** Densitometric analysis of relative protein level as shown in Figure 7c. **c,** Densitometric analysis of relative protein level as shown in Figure 7d.

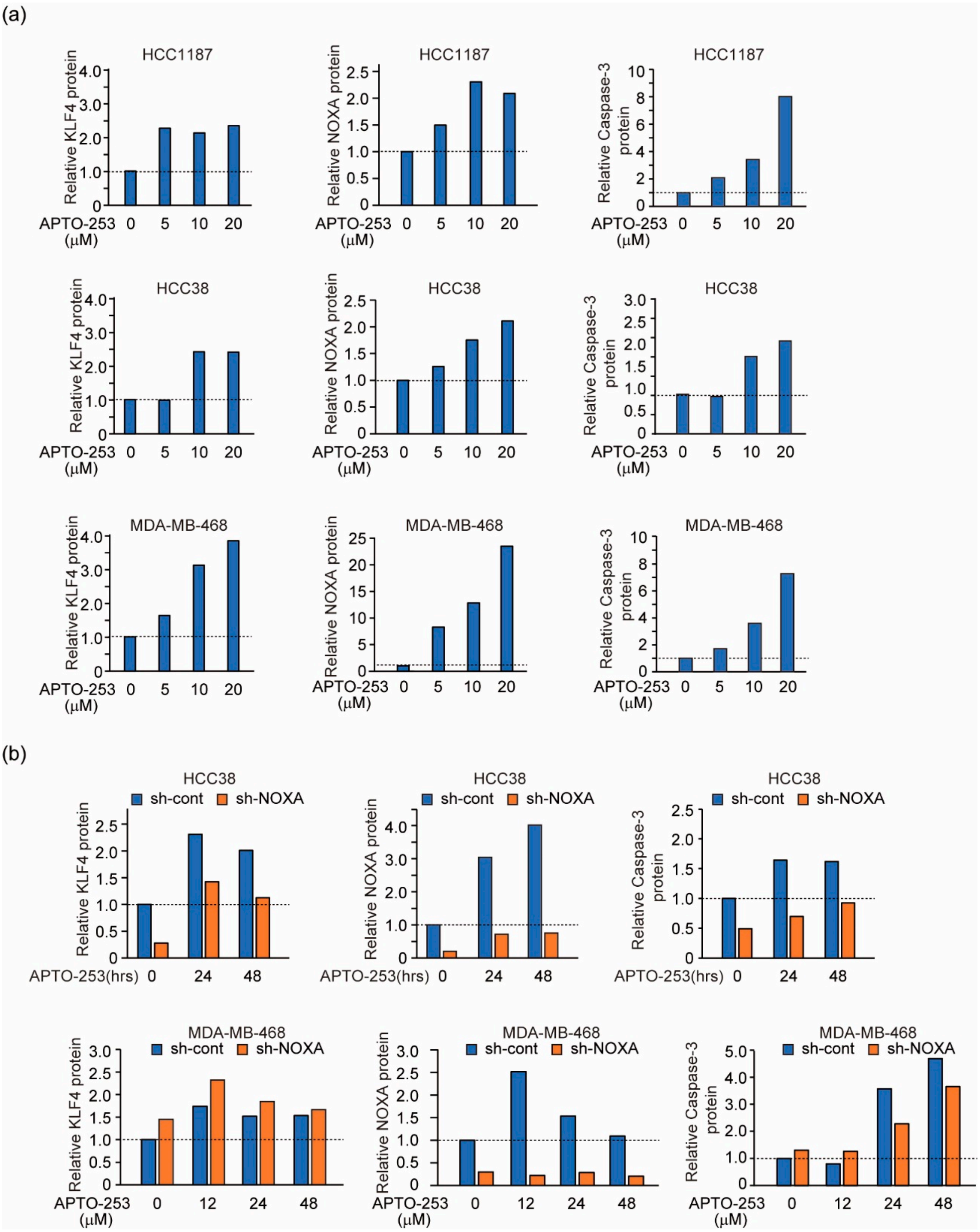

Figure S6. Densitometric analysis of relative protein level as shown in Figure 8. Indicated protein levels were normalized to total loading control protein. **a,** Densitometric analysis of relative protein level as shown in Figure 8b. **b,** Densitometric analysis of relative protein level as shown in Figure 8c.
